# A comparison of static vs dynamic surface topography measurements in the evaluation of scoliosis

**DOI:** 10.1186/1748-7161-7-S1-P12

**Published:** 2012-01-27

**Authors:** P Knott, K Smith, L Mack, L Peters, N Patel, S Thompson, S Mardjetko

**Affiliations:** 1 Rosalind Franklin University North Chicago, USA; 2 Illinois Bone and Joint Institute, USA

## 

The Formetric 4D System (Diers International GmbH, Schlangenbad, Germany) is a surface topography system used to evaluate trunk deformity in patients with scoliosis. The original system took a single static scan of the patient which was subject to variability due to the natural sway of the body during standing. A 4D system was developed that takes multiple scans over 6 seconds and averages them to take into account patient movement during imaging. Averaged scans such as this should show less variability over time than single images do [1-4].

The purpose of this study was to compare surface topography measurements taken as a single scan with those taken as averaged scans over 6 and 15 seconds to see whether the averaged scans have less variability.

Six volunteer patients without scoliosis were used to measure the position of normal spines in the standing position. Each patient was scanned ten times for each time interval. For each group of scans, a standard error of measurement was calculated. The patients were then compared based on the standard errors for each topography parameter. Data included 6 patients x30 measurements each x10 topography parameters totaling 1800 data points.

Data analysis was done using ANOVA to compare the three groups. There were not significant differences between the groups in any of the surface topography parameters that were studied. Our conclusion was that in this population of 6 normal patients, we were not able to demonstrate smaller standard errors of measurement with averaged 4D scans than with single scans.
